# Malignant Degeneration of a Mature Ovarian Teratoma

**DOI:** 10.1155/2021/5527467

**Published:** 2021-07-22

**Authors:** Mariam Mahtate, Sarah Talib, Aziz Slaoui, Najia Zeraidi, Amina Lakhdar, Brahim Rhrab, Aziz Baydada

**Affiliations:** ^1^Gynecology-Obstetrics and Endoscopy Department, Maternity Souissi, University Hospital Center IBN SINA, University Mohammed V, Rabat, Morocco; ^2^Gynecology-Obstetrics and Endocrinology Department, Maternity Souissi, University Hospital Center IBN SINA, University Mohammed V, Rabat, Morocco

## Abstract

Mature cystic teratoma is the most common type of ovarian germ cell neoplasm, but occasionally, it can undergo malignant transformations, especially in postmenopausal women. These secondary malignant neoplasms are most commonly squamous cell carcinomas. The absence of clinical and radiological specificity of this transformation means that the diagnosis remains purely histological. Data is insufficient regarding the appropriate management given their rarity. However, the treatment is multidisciplinary and is based on surgery and a platinum-based chemotherapy regimen. We report the case of a 53-year-old postmenopausal female patient with malignant transformation of the ovarian teratoma who was treated surgically and whose outcome was favorable. The diagnosis of the teratoma was evoked on imaging, while the diagnosis of squamous cell carcinoma was revealed on histology. Malignant transformation is an uncommon complication of mature ovarian teratomas. No clinical, radiological, or biological sign is specific; therefore, resection of any ovarian mass, even asymptomatic, is required.

## 1. Introduction

Mature cystic teratomas (MCT) of the ovary, commonly known as dermoid cysts, are the most common type of ovarian germ cell neoplasms (10 to 20%). They occur mainly in young women of childbearing age [1, 2].

Malignant transformation (MT) of MCT is a rare complication, with an estimated incidence of less than 2% [1, 2]. Most often seen in the postmenopausal period, it corresponds to the transformation of one of the components of the dermoid cyst into a cancerous tissue of a nongerminal nature which can be an epidermoid carcinoma, adenocarcinoma, or exceptionally a sarcoma or melanoma [2–4]. Squamous cell carcinoma (SCC) is the most frequent malignant degeneration arising from the ectodermal component of MCT. Their clinical presentation is nonspecific and varies according to the tumor stage and is similar to that of benign ovarian cysts. The diagnosis is established by the histological study of the surgical piece [5].

We report the case of a 53-year-old postmenopausal female patient with malignant transformation of the ovarian teratoma who was treated surgically and whose outcome was favorable. The diagnosis of the teratoma was evoked on imaging, while the diagnosis of squamous cell carcinoma was revealed on histology.

## 2. Case Presentation

**Figure 1:**
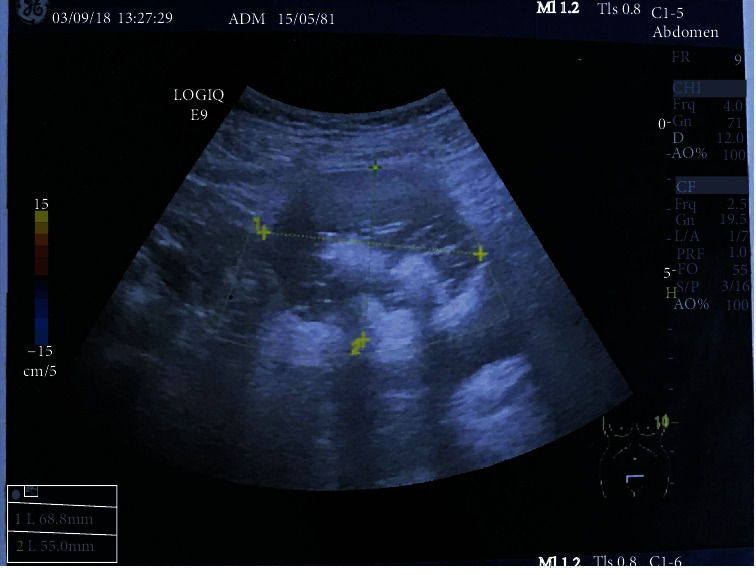
Pelvic mass of 68 × 55 mm which had the features of a dermoid ovarian cyst with a heterogeneous echotexture and presence of internal echoes with high echogenicity.

A 53-year-old female gravida 3, para 3, with no specific medical history, being in menopause for the past 6 years, presented in our department for an increase in abdominal volume and pelvic pain evolving for 4 months. The physical and gynecological examination revealed a palpable and painful mass on the right abdominal wall. The ultrasound images revealed the presence of a pelvic mass of 68 × 55 mm which had the features of a dermoid ovarian cyst (Figure 1). Pelvic magnetic resonance imaging (MRI) showed a round, well-defined right ovarian mass of approximately 63 × 53 × 61 mm of suprauterine location, with intratumoral fat-like signal intensity, which indicates a mature cystic teratoma. Tumor markers were within the normal range with cancer antigen 125 (CA 125) at 13.1 UI/mL and CA 19-9 at 20 UI/mL.

**Figure 2:**
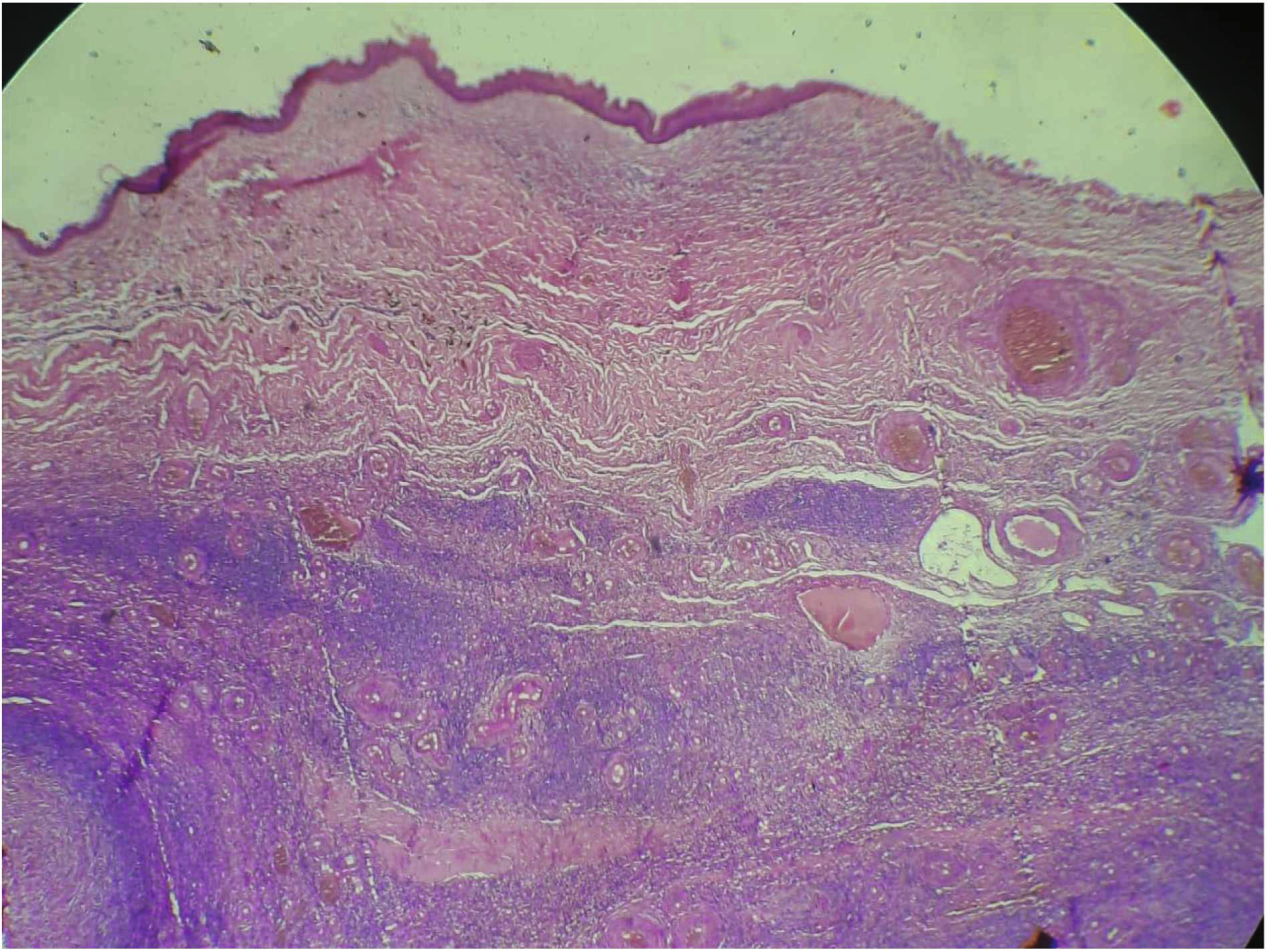
Cystic wall formed by a hyalinized fibrous ovarian stroma in which the covering is respiratory type with the presence of mature glial tissue; hematoxylin and eosin staining, ×40.

**Figure 3:**
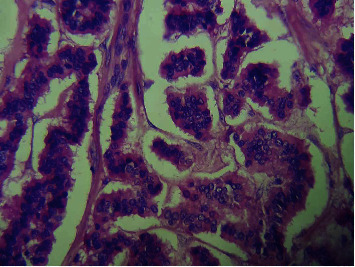
High cell density tumor proliferation made up of anastomotic cord intercepted by monomorphic clusters with rounded nuclei or ovoid granular chromatin without mitotic activity; hematoxylin and eosin staining, ×400.

A decision for laparotomy evaluation was made. The surgical exploration found an ascites of low abundance and a cystic mass adhering to the right ovary. The frozen section diagnosis of the mass returned in favor of a squamous cell carcinoma arising in mature cystic teratoma without infiltration or rupture of the ovarian capsule (Figures 2 and 3). It was then decided to perform a total hysterectomy with bilateral salpingo-oophorectomy and omentectomy. The patient was discharged from the hospital on the second day with an uneventful postoperative course. After a multidisciplinary meeting, adjuvant chemotherapy was decided (bleomycin, etoposide, and cisplatin (BEP) regimen).

## 3. Discussion

Malignant transformation of mature teratomas is defined as the development of carcinoma on one of the mature components of the dermoid cyst. It is an uncommon complication that arises in less than 2% of patients [3]. This complication occurs most often during the postmenopausal period [2–4]. The age of onset of this degeneration in our patient was 53 years old, which is consistent with the literature with an average age of onset of 54 years old reported by several authors [2–4].

These secondary malignant neoplasms are most commonly squamous cell carcinomas. They are derived from the ectoderm [6]. The diagnosis is made postoperatively, after surgical treatment of a supposedly benign dermoid cyst. The clinical presentation of cysts including heaviness and pelvic pain is nonspecific, varies according to the tumor stage, and is similar to that of benign ovarian cysts. In advanced cancers with metastases, ascites and urinary and digestive symptoms are usually the cause of the diagnosis [5].

While ultrasound is the radiological examination of choice in the diagnosis and monitoring of mature teratomas, it does not detect signs of malignant transformation [7]. Indeed, ultrasound diagnosis suggests the common elements of dermoid cysts, which are a solid organic formation, containing an echogenic focus with distal acoustic attenuation or shadow cone (related to the presence of the Rokitansky nodule in the cystic cavity), formation containing hair, teeth, calcifications, and other atypical, hyperechoic, nonvascularized tissues [7]. The fatty and sebaceous contents are best visualized on MRI and pelvic-abdominal CT scan [8]. Imaging diagnoses most of the time a presumably benign dermoid cyst except when there is an advanced cancer with penetration of the ovarian capsule and local spread [7, 8]. Some authors also suggest the following as signs of malignancy: adhesion to neighboring structures, the presence of nodules, increased wall thickness, and the presence of necrosis and hemorrhage [7, 8]. Furthermore, the malignancy can also be suspected on intraoperative criteria such as age greater than 40 years, large tumor size which can reach 20 cm, and the presence of hemorrhage and necrosis [1]; but it is only the histology study that confirms the degeneration of the MCT. In our case, we relied on the ultrasound and MRI results to suggest the diagnosis of mature teratoma but it is the frozen section that confirmed the diagnosis of a squamous cell carcinoma arising in mature cystic teratoma.

The tumor markers used to help characterize ovarian lesions are not very specific and cannot be used to differentiate malignant from benign ovarian tumors like in our case. Squamous cell carcinoma (SCC) antigen may be increased in transformed MCT associated with squamous cell carcinoma [8, 9]. However, a low level of SCC antigen does not formally rule out a cancerous teratoma [9]. CA 125 is a glycoprotein secreted by the majority of serous ovarian tumors; it is used to assess sensitivity to chemotherapy and for the diagnosis of recurrences. Therefore, CA 125 has a diagnostic, prognostic, and therapeutic evaluation value [9]. Alpha-fetoprotein (AFP) should not be interpreted as a factor of malignancy, its production being determined by the endoderm tissue of the dermoid cyst. CA 19-9 may be elevated in malignant and benign tumors [9]. In our case, tumor markers were within the normal range. Hackethal et al.'s meta-analysis on 277 cases of squamous cell carcinoma arising in cystic teratoma found high SCC antigen in 86.5% of cases, high CA 125 in 71% of cases, high CA 19-9 in 77% of cases, and ACE present in 40% of cases [9].

The diagnosis of dermoid cyst is confirmed during surgery on the macroscopic appearance with a solid ovarian cyst containing fat, skin, hair, and teeth. In most cases, the diagnosis of malignant transformation is a surprise given by the histology [8].

Surgical treatment of these malignant transformations is the same as for ovarian carcinoma: laparotomy in the majority of cases with unilateral or bilateral adnexectomy. A second complete exploration if the frozen section was not initially performed is necessary to respect the rules of oncology with systematic peritoneal cytology, biopsies of suspicious areas, omentectomy, total hysterectomy, and pelvic and para-aortic lymphadenectomy. Chemotherapy using alkylating agents improves prognosis for advanced stages, but not the use of radiotherapy [5–7]. In the case of young patients who wish to maintain their fertility, having a transformed dermoid cyst limited to the ovarian capsule, without local or distant invasion, conservative treatment may be possible: cystectomy or unilateral adnexectomy with multiple peritoneal biopsies. For elderly patients, the treatment is as for any ovarian cancer: total hysterectomy with bilateral adnexectomy, omentectomy, and lymphadenectomy [6–9]. Our patient underwent total hysterectomy with bilateral salpingo-oophorectomy and omentectomy.

The prognosis depends on the stage, the presence of vascular invasion, and the rupture of the ovarian capsule [10]. To establish a prognosis, Kikkawa et al. [10] also take into consideration the presence or absence of tumor residue; thus, the 5-year survival is 79% without tumor residue and 10.1% with tumor residue.

## 4. Conclusion

Occurring preferably in the postmenopausal period, malignant transformation of mature cystic teratoma is a well-known but uncommon phenomenon. There is currently no formal diagnostic criterion before the histological analysis. An attempt is made to adapt the surgical aggressiveness according to the age of the patient. Conservative surgery is reserved for young women, especially nulliparous who wish to preserve fertility. Hysterectomy and bilateral salpingo-oophorectomy are advised in postmenopausal patients.

## Data Availability

Supporting materials are available if further analysis is needed.

## Abbreviations

MCT: Mature cystic teratoma
SCC: Squamous cell carcinoma
MT: Malignant transformation
MRI: Magnetic resonance imaging
CA 125: Cancer antigen 125
CA 19-9: Cancer antigen 19-9
AFP: Alpha-fetoprotein
BEP: Bleomycin, etoposide, and cisplatin chemotherapy regimen.
